# Modeling Fluid Resuscitation by Formulating Infusion Rate and Urine Output in Severe Thermal Burn Adult Patients: A Retrospective Cohort Study

**DOI:** 10.1155/2015/508043

**Published:** 2015-05-10

**Authors:** Qizhi Luo, Wei Li, Xin Zou, Yongming Dang, Kaifa Wang, Jun Wu, Yongqin Li

**Affiliations:** ^1^Burn Research Institute, Southwest Hospital, State Key Lab of Trauma, Burn and Combined Injury, Third Military Medical University, Chongqing 400038, China; ^2^School of Biomedical Engineering, Third Military Medical University and Chongqing University, Chongqing 400038, China

## Abstract

Acute burn injuries are among the most devastating forms of trauma and lead to significant morbidity and mortality. Appropriate fluid resuscitation after severe burn, specifically during the first 48 hours following injury, is considered as the single most important therapeutic intervention in burn treatment. Although many formulas have been developed to estimate the required fluid amount in severe burn patients, many lines of evidence showed that patients still receive far more fluid than formulas recommend. Overresuscitation, which is known as “fluid creep,” has emerged as one of the most important problems during the initial period of burn care. If fluid titration can be personalized and automated during the resuscitation phase, more efficient burn care and outcome will be anticipated. In the present study, a dynamic urine output based infusion rate prediction model was developed and validated during the initial 48 hours in severe thermal burn adult patients. The experimental results demonstrated that the developed dynamic fluid resuscitation model might significantly reduce the total fluid volume by accurately predicting hourly urine output and has the potential to aid fluid administration in severe burn patients.

## 1. Introduction

Accounting for nearly 330,000 deaths per year, acute burn injuries are among the most devastating forms of trauma and lead to significant morbidity and mortality in the world [1, 2]. Thermal injury disrupts normal homeostasis and results in release of inflammatory mediators that increase capillary permeability and lead to fluid leaking from the circulation into interstitial space and to evaporation [3–5]. Failure to correct the fluid losses will result in decreased cardiac output, acute renal failure, vascular ischemia, cardiovascular collapse, and even death [6]. Appropriate fluid resuscitation after severe burn, specifically during the first 48 hours following injury, is therefore considered as the single most important therapeutic intervention in burn treatment [7].

Many fluid resuscitation formulas/protocols have been introduced as guidelines of fluid management when the total body surface area (TBSA) involved in the burn approaches is 20%, such as the Parkland, modified Parkland, Brooke, modified Brooke, Evans, Muir-Barclay, Monafo, Haifa formula, and the Third Military Medical University (TMMU) protocol [5, 8, 9]. These formulas give the recommended infusion volume and rate required to resuscitate a patient based on the body weight and TBSA burned. Although the absolute consensus on resuscitation formula has not been reached, an important issue exempt from debate is that the formulas are used only as a starting point for fluid resuscitation because each patient reacts differently to burn injury and resuscitation [10]. As a result, the fluid administration schemes do not exclusively follow fixed formulas but are regularly adjusted based on patient's clinical signs of adequate organ perfusion, as inferred from urine output (UOP) of 0.5–1.0 mL/kg/hr [11].

The phenomenon of insufficient fluid related under-resuscitation has greatly reduced with the advances in prehospital care and burn resuscitation training over the past decades. However, there is growing evidence that patients with major burns received far more fluid than the formulas recommend [12–14]. Over-resuscitation, which is known as “fluid creep,” has emerged as one of the most important problems during the initial period of burn care [14–17]. Two meta-analysis of fluid requirements for burn injury both showed that the total volume administrated was exceeding the formulas estimate and the mean UOP was above the high end of target level [18, 19]. Just as inadequate fluid resuscitation can lead to hypovolaemic shock, organ failure and systemic inflammatory response syndrome and excessive fluid resuscitation may result in increased risk of infectious complications, acute respiratory distress syndrome, abdominal compartment syndrome, and even death [10, 12, 19–23].

The fine balance between too little or too much fluid is hard to maintain and requires clinicians with extensive burn experience. Many clinicians complained that current formulas were too cumbersome to follow [24]. Therefore, if fluid titration can be personalized and automated during the resuscitation phase, more efficient burn care and outcome will be anticipated.

In this retrospective study, a dynamic fluid resuscitation model was developed by formulating UOP and infusion rate based on the observational data recorded during the initial 48 hours after injury from severe adult burn patients. The model was then validated in another population of burn patients. We hypothesized that the UOP prediction based fluid titration model could offer reliable fluid management.

## 2. Materials and Methods

### 2.1. Data Collection

This study was approved by the ethics committee of Southwest Hospital. The informed consent was waived by the committee because this was a retrospective and observational study and the data were analyzed anonymously. Patients admitted to the Burn Research Institute of Southwest Hospital between January 2011 and December 2013 were enrolled. Inclusion criteria for this study were all patients who presented within 12 hours following thermal injury, with TBSA burned greater than 30%, and adult patient whose age ranged from 16 to 60 years old. Those with electrical or chemical burns and those with combined injuries such as inhalational injury, acute kidney injury, congestive heart failure, trauma or blood loss, alcohol intoxication and using any diuretic, sedation or with mechanical ventilation, and escharotomy/surgery during the initial 48 hours were excluded.

Patients were resuscitated according to the TMMU protocol, which was developed in the 1960s and is the most widely used for fluid resuscitation in China for treating patients with severe burns [9, 25, 26]. This protocol suggests that, for adult burn patients, 1 mL of lactated Ringer's solution and 0.5 mL of plasma per percent TBSA burn area and per kilogram body weight (mL/kg/%TBSA) are given in the first 24 hours following injury. In addition, 2 L water (as a 5% glucose solution) was added as a daily basic requirement for adults. During the first 24 hours, half of the calculated fluid should be administered within the first 8 hours after burn and the remainder is homogeneously administered in the following 16 hours. During the next 24 hours, after injury either for adults or children, the protocol recommends that half of the amount of crystalloid solution and colloid of that used during the first 24 hours should be administered, and 2 L water should be given. The infusion rate was adjusted hourly according to UOP measurement. If UOP was less than 30 mL/hr or greater than 60 mL/hr, a 20–30% increase or decrease was administered.

The fluid was administrated through infusion pump (TE-171, TERUMO Corporation, Tokyo, Japan) and the UOP was collected with scaled urine bag (SL125, Senlin Medical Products, Changshu, China) during the postburn resuscitation period. Clinical data, including hourly infusion rate and UOP, were prospectively collected by trained nurses and validated by independent physician. Data of 2011 was served as derivation, while data of 2012 and 2013 was used to validate the proposed model.

### 2.2. Model Development

**Figure 1:**
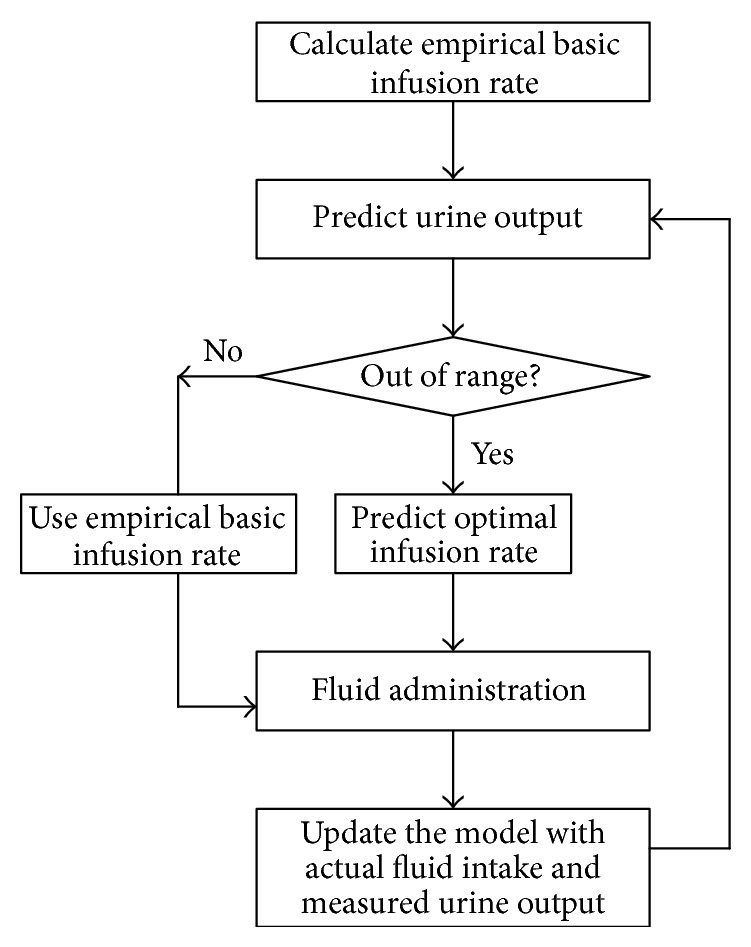
Flowchart of the proposed fluid management model.

The flowchart of the proposed dynamic fluid management model is shown in Figure 1. The defined variables and the specific equations used to calculate these parameters are listed in Table 1. Average infusion rates at each hour after burn were analyzed in the derivation group to determine the basic infusion rate for this cohort. A set of curve fit methods was used to derive the empirical infusion rate. Potential factors such as body weight and TBSA burned that might affect the model were used as candidates to adjust the basic infusion rate. The adjusting factor was defined as the average infusion rate of each patient during the initial 48 hours divided by the average infusion rate of all patients in derivation data set.

The UOP prediction model comprised of two major parts. One part was the overall response of total fluid on urine production, calculated as total UOP divided by all fluid infused. Another part was the instant effect of current hour's fluid infusion on UOP, represented as ratio of hourly infusion to measured UOP. A set of linear regression methods was used on both the long term and short term response of fluid infused to predict next hour's UOP and, therefore, to evaluate patient's response before infusion.

The empirical infusion rate would be retained if predicted UOP was within the accepted target range. Otherwise, an optimal infusion rate would be given according to the UOP prediction model. Current hour's actual fluid intake and UOP were then used to update the parameters for next hour's prediction.

### 2.3. Evaluation of the Proposed Model

The proposed model was evaluated using the validation dataset. For purpose of UOP prediction, the predicted UOP was compared with measured UOP at each hour for each patient. The absolute and relative absolute prediction errors were calculated and reported. Absolute prediction error was defined as the absolute value between the predicted and actual UOP, and relative absolute prediction error was defined as absolute prediction error divided by actual UOP. Sensitivity, specificity, and accuracy for predicting over range UOP were also calculated since the aim of UOP prediction was to identify potential improper fluid managements. Sensitivity was defined as the proportion of UOP beyond the accepted range and the prediction was positive for it. Specificity was defined as the proportion of UOP that was within the accepted range and the prediction was negative for it. Accuracy was the probability to obtain a correct prediction. For purpose of infusion rate prediction, comparisons between the experimental and theoretical results, including the total infusion fluid volume and average infusion rate over initial and second 24 hours, were performed. The comparative results were used to assess whether the infusion rate prediction model can reduce the total fluid volume and resuscitation ratio or prevent the excessive fluid infusion.

### 2.4. Statistical Analysis

Curve estimation and regression analysis were used to estimate the parameters of the prediction model and adjust function. Pearson coefficient of correlation was used to illustrate the relation between potential adjusting variable and adjusting factor. In order to compare the means between experimental and theoretical results, paired-samples *t*-test was used for analysis. A two-sided probability value less than 0.05 was considered significant.

## 3. Results

During the investigated period, 5,240 patients presented to the institute and 37 of them met the inclusion criteria. Thirteen patients were administrated in 2011, 10 in 2012, and 14 in 2013. The average burn area was 51.6% TBSA, with a minimum of 30.2% and a maximum of 97.0%. The demographic and fluid resuscitation data are presented in Table 2. No significant differences were observed between groups in terms of age, body weight, TBSA burned, area of full thickness burn, total fluid infused, and total UOP measurement. A total of 541 patients' hours data, including hourly infusion rate and UOP, were available for model development. At the same time, a total of 975 patients' hours data were captured for validation.

**Figure 2:**
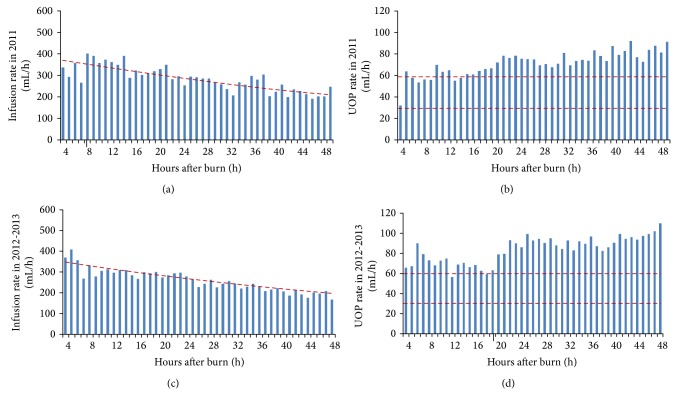
Average infusion and urine output (UOP) rates in derivation and validation dataset.

There were no obvious difference between the total fluid intake and TMMU protocol suggested volume over the initial 48 hours (11.0 ± 3.0 versus 11.8 ± 2.9 L, *p* = 0.236 for derivation; 10.1 ± 3.4 versus 10.8 ± 2.6 L, *p* = 0.143 for validation).  Figure 2 shows the average fluid intake and measured UOP per hour during the resuscitation period. Both the derivation and validation data showed similar patterns; that is, the infusion rate is decreasing while UOP rate is increasing over time. The average fluid administrated during the second 24 hours was significantly lower than the initial 24 hours for patients in 2011 (227.7 ± 52.3 versus 307.9 ± 65.4 mL/hr, *p* < 0.001) and in 2012-2013 (216.3 ± 26.1 versus 301.5 ± 35.2 mL/hr, *p* < 0.001). The average UOP, on the other hand, was markedly higher during the second 24 hours compared with that of the first 24 hours in both derivation (78.2 ± 24.1 versus 65.8 ± 29.9 mL/hr, *p* = 0.026) and validation groups (93.6 ± 6.5 versus 73.0 ± 10.7 mL/hr, *p* < 0.001). The calculated lower limit was 30.9 ± 4.8 mL/hr and the upper limit was 61.9 ± 9.6 mL/hr in this population based on the recommended 0.5–1.0 mL/kg/hr UOP to ensure adequate resuscitation.

### 3.1. Basic Infusion Rate Model and Adjusting Function

The pattern of overall fluid infused per hour indicated a continuous decay pattern over the entire 48 hours. Experimental results demonstrated that an exponential decay model with the following format has the best fit for the infusion rates:

(1) $$\begin{array}{c} \text{Rate}\left(\;t\;\right)=a_{1}*e^{-a_{2}*t}. \end{array}$$

Here, *a*_1_, *a*_2_ represented the function coefficients of decay and *t* was hour after burn.

The correlation coefficient was 0.888 between TBSA burned and adjusting factor (*p* < 0.001) and −0.267 between weight and adjusting factor (*p* = 0.378). TBSA burned was therefore used as a modifier and the following modifier function was obtained by regression analysis:

(2) $$\begin{array}{c} \text{Adjust}\_\text{TBSA}=b_{1}+b_{2}*\text{TBSA}. \end{array}$$

The basic infusion rate is then presented as

(3) $$\begin{array}{c} \text{Base}\_\text{Rate}\left(\;t\;\right)=\text{Rate}\left(\;t\;\right)*\text{Adjust}\_\text{TBSA}. \end{array}$$

The basic infusion model was evaluated with the average infusion rates in validation dataset. No significant difference was observed when the hourly predicted basic infusion rate was compared with the actual intake (261.8 ± 44.6 versus 256.1 ± 52.6 mL/hr, *p* = 0.08). The mean absolute prediction error was 15.8 ± 15.3 mL/hr and the relative absolute prediction error was 6.4 ± 6.0%.

### 3.2. UOP Prediction Model

**Figure 3:**
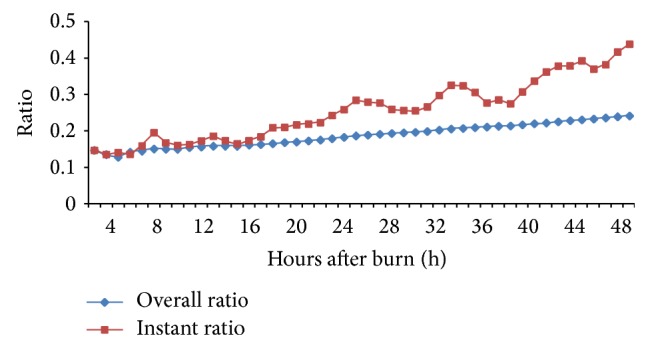
Overall and instant urine production ratios obtained from the derivation dataset.

Figure 3 shows the overall ratio and instant ratio obtained from derivation dataset. The two ratios reflecting the long term and short term effects of fluid infused on urine production were computed as

(4) Overall_Ratiot∑0tUOPt∑0tFluid_Ratet,

(5) Instant_Ratiot=UOPtFluid_Ratet,

where UOP(*t*) and Fluid_Rate(*t*) represented the hourly measured UOP and actual fluid intake by the end of time *t*. Since UOP(*t*), Fluid_Rate(*t*), Overall_Ratio(*t*), and Instant_Ratio(*t*) were known as parameters at the beginning of hour *t* + 1, the coming hour's UOP could be estimated with a regression model based on Base_Rate(*t* + 1) that was obtained from the basic infusion rate model (equation (3)):

(6) UOP_Predt+1=c1∗Overall_Ratiot−1∗∑0tFluid_Ratet−∑0tUOPtt+c2∗Instant_Ratiot∗Base_Ratet+1+Pred_Errt+1.

Coefficients *c*_1_, *c*_2_ are constant and Pred_Err(*t* + 1) denoted the predict error that was recursively estimated from previous hour's prediction error and rate change of UOP:

(7) Pred_Errt+1=d1∗UOP_Predt−UOPt+d2∗UOPt−UOPt−1.

These two parameters were selected because correlation analysis showed that current hour's prediction error was linearly correlated with previous hour's prediction error (*r* = 0.409; *p* = 0.005) and rate change in UOP measurement (*r* = −0.391; *p* = 0.007).

**Figure 4:**
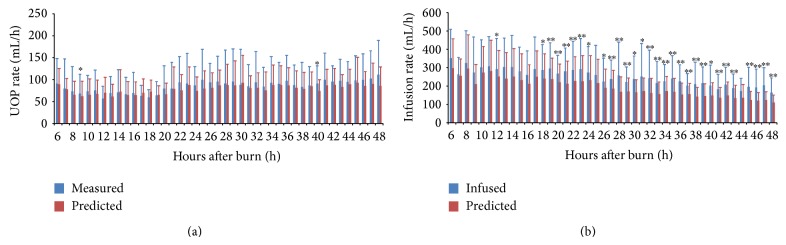
(a) Average urine output (UOP) between experimental and predicted values for patients in validation. (b) Average infusion and predicted fluid rate with validation dataset. ^∗^ *p* < 0.05; ^∗∗^ *p* < 0.01.

Because the model requires the previous 2 hours' data to predict the following hour's UOP, therefore, a total of 927 predictions was performed using validation dataset. There were no significant differences (Figure 4(a)) between hourly UOP measurement and prediction, except for a relative lower prediction at 10 (68.2 ± 44.0 mL/hr versus 61.7 ± 34.4 mL/hr, *p* = 0.047) and 40 (91.1 ± 40.3 mL/hr versus 73.8 ± 26.1 mL/hr, *p* = 0.033) hour after burn when paired *t*-test was performed. The absolute prediction error was 23.9 ± 7.7 mL and the relative absolute error was 29.8 ± 5.6% over the entire resuscitation period. The sensitivity, specificity, and accuracy for prediction of over range UOP were 85.2%, 65.0%, and 77.0% when target UOP was defined as 30–60 mL/hr.

### 3.3. Infusion Rate Prediction Model

The combination of the hourly basic infusion rate estimation and UOP prediction algorithm resulted in the final fluid management model. If the predicted hourly UOP was greater than the upper limit or less than the lower limit of target, the infusion rate would be replaced with the following equation:

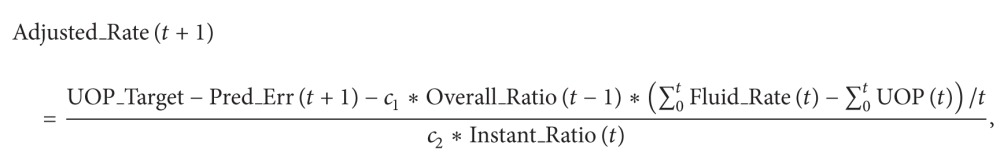
(8)where UOP_target was the mean value of lower and upper limit of the expected target UOP. Otherwise, if the predicted UOP was within the accepted range of target settings, the infusion rate computed from the base rate model would be retained:
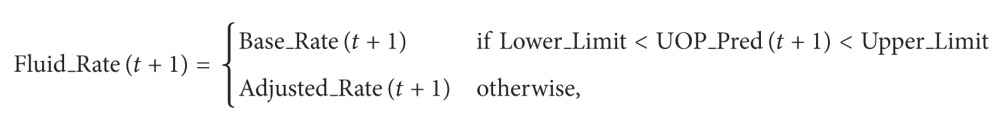
(9)where Lower_Limit and Upper_Limit were the lower and upper limit value of the target UOP.

The notional infusion rate and overall fluid required were calculated using the final fluid management model for the 24 patients in 2012-2013. Instead of using Base_Rate(*t*), the actual infusion rate was used when the predicted hourly UOP was within the target level for purpose of validation.  Figure 4(b) illustrates the actual and predicted infusion rate when the target UOP was set to 30–60 mL/hr. The predicted infusion rate could be obviously decreased in 42.1% of the time during the first 24 hours (258.4 ± 48.3 mL/hr versus 301.5 ± 35.2 mL/hr, *p* < 0.001) and 91.7% of the time during the second 24 hours (155.1 ± 24.6 mL/hr versus 216.3 ± 26.1 mL/hr, *p* = 0.001). The notional infusion volumes might be significantly reduced during the initial 24 hours (4.1 ± 1.7 L versus 4.9 ± 2.1 L, *p* = 0.003) and would be further dropped during the second 24 hours (3.7 ± 1.2 L versus 5.2 ± 1.8 L, *p* < 0.001) after burn.

## 4. Discussion

In the present study, a dynamic fluid management model combining hourly infusion rate estimation and UOP prediction was developed and evaluated with separate datasets. The major findings are as follows: (1) hourly mean UOP was significantly higher during the second 24 hours compared with that of initial 24 hours after burn, even though the infusion rate was relatively lower with the use of TMMU protocol; (2) the UOP prediction model gave the reasonable explanation of the increased UOP during the second 24 hours and could reliably predict the potential over range UOP production during resuscitation period; (3) the fluid management model might dramatically reduce the fluid required for resuscitation, especially during the second 24 hours after injury.

In order to understand the mass exchange following thermal burn, several mathematical models that described the distribution and exchange of fluid and solutes following the injury have been proposed and tested [27–30]. These mass exchange models are helpful in understating the complex physiological changes, the implications of different fluid resuscitation regimens, and the mechanistic effects of drugs. However, the complexity of these models hindered their potential clinical applications. To uniform resuscitation and optimize fluid titration, several infusion rates decision and control algorithms have been developed and investigated [19, 31–33]. Bowman and Westenskow [31] introduced a microcomputer-based fluid infusion system for fluid resuscitation with real-time measurement of infused fluid and UOP. The system controlled the fluid infusion with a proportional-integral-derivative (PID) algorithm. Salinas et al. [19] developed an automated fluid balance monitor system based on the PID model. In a recent clinical study, Salinas et al. [33] evaluated the efficacy of a computer decision support system (CDSS) that integrated PID model, decision-assist and closed-loop algorithms. Although the required infusion volume was statistically decreased and the outcome was significantly improved in CDSS group compared to historical cohort, Staff members were not yet convinced of a positive correlation between CDSS technology and patient outcomes. A neutral response to the questions regarding trust in recommendation satisfied with the system not only reflected the potential disconnection existed between the success of new technology and trust in recommendation by the CDSS but also revealed that the underlying models used for fluid management merits further validation [34].

In the current study, we proposed a refined fluid management model based on the mass exchange and the fluid response models. The model firstly calculates hourly empirical infusion rate according to an exponential decay equation. The result was consistent with Salinas' report, even though a different resuscitation protocol was used [33]. In order to compensate intrapatient variations in burned TBSA and body weight, an adjusting factor was defined and correlated with burned TBSA and body weight. Interestingly, statistical analysis showed that burned TBSA was a significant modifier while body weight was not. This may be due to the relative small standard deviation in body weight for this population. But it can be easily incorporated into the model if a wide range of population's body weight is considered. In order to obtain an optimal outcome and adjust the infusion rate accordingly, a UOP prediction model was developed to predict next hours' UOP using empirical infusion rate, overall, and instant urine production ratios. The overall ratio that was computed from the total amount of UOP measured and the amount of fluid infused after injury is assumed to reflect the urine produced by the total blood available in the circulatory system following a burn injury based on the mass exchange model [30]. On the other hand, the instant ratio that was calculated through previous hour's fluid intake and UOP measurement may reflect the immediate effect of fluid infusion on urine production according to the fluid response model [19, 33]. These assumptions were physiologically reasonable and more importantly the increased instant ratio could explain the increased hourly UOP during the seconds 24 hours compared with that of initial 24 hours, even though a relatively lower infusion rate was administrated. Additionally, the introduction of error estimation for UOP prediction by previous hour's prediction error and rate change of UOP might compensate the effects of fluid losses via exudation and evaporation on urine production. Since the accuracy of UOP prediction plays an important role in this fluid management model, we therefore validated the performance of the UOP prediction algorithm with the 927 patient hours' data. When the lower limit was set to 30 mL/hr and the upper limit was set to 60 mL/hr, the overall accuracy was 77.0% and 85.2% patient hours' over range UOP could be accurately predicted. Another important refinement of the fluid management model was that the predicted UOP served as judger for next hour's infusion rate adjustment. If the predicted UOP was within the accepted range, such as 30–60 mL/hr or 0.5–1.0 mL/kg/hr, the empirical infusion rate will be unchanged. But if the predicted UOP was out of the accepted range, the infusion rate would be adjusted according to the UOP prediction model. Excessive fluid infusion, especially during the second 24 hours, might be avoided accordingly. However, we noticed that the correlation between the calculated and estimated UOP and infusion rates was better in the initial 24 hours than later as the resuscitation advances. The second 24 hours might be related to the period that patients entered the diuretic phase of burn resuscitation and urinary output was not always driven by hourly infusion rates during the period of fluid mobilization after active resuscitation was complete. Therefore, defining the end point of active resuscitation for each patient and modeling fluid resuscitation and mobilization as separate entities could be a practical solution to improve the predictive value for fluid therapy.

We recognized that several limitations need to be considered in the study. Firstly, the patients were resuscitated based on TMMU protocol; the effect of different fluid resuscitation formulas on the predictability of UOP needs to be investigated. Secondly, measurement error introduced during manual recording might affect the overall performance of prediction. We anticipated that using automatic infusion pump and urine meter measurement would improve the prediction accuracy. Thirdly, patients with acute kidney injury, inhalation injury, trauma, diuretic or sedation administration, and utilization of mechanical ventilation were excluded in the study. The effect of these comorbid conditions and circumstances on the prediction of urine output was still unknown and will be investigated in our future studies. Additionally, whether utilization of hemodynamic monitoring, such as heart rate and mean arterial pressure into the model, could improve the performance of the model has not been investigated. Fourthly, this was a retrospective study; whether the total amount of fluid infused could be decreased and whether more efficient resuscitation could be achieved using the proposed fluid management model still require further validation.

## 5. Conclusions

In this retrospective study, over range UOP was mainly observed during the second 24 hours after injury. Over range UOP could be reliably predicted and the total fluid volume might be significantly reduced using the dynamic resuscitation model. The proposed model therefore has the potential to aid fluid administration in severe burn patients.

## Figures and Tables

**Table 1:** Variables, definitions, and related equations for the proposed model.

Variable	Definition	Equation
Basic infusion rate	The empirical infusion rate that was estimated by time of postburn and total body surface area burned.	(3)

Overall ratio	Total urine output divided by all fluid infused by the end of time *t*.	(4)

Instant ratio	Ratio of urine output rate and infusion rate at time *t*.	(5)

Predicted urine output	The predicted hourly urine output at the beginning of time *t* + 1.	(6)

Adjusted rate	The predicted infusion rate according to mean value of lower and upper limit of the expected target urine output.	(8)

Fluid rate	The suggested infusion rate.	(9)

**Table 2:** The demographic and fluid resuscitation data [mean ± SD].

	Derivation (*N* = 13)	Validation (*N* = 24)	*p* value
Age (years)	40.8 ± 13.0	42.2 ± 14.5	0.766
Male (*N*)	12 (92.3%)	17 (70.8%)	0.116
Body weight (kg)	61.5 ± 9.4	61.9 ± 9.6	0.915
Starting time (hrs)	7.2 ± 4.5	8.3 ± 4.6	0.459
Total burn area (%TBSA)	56.5 ± 20.9	49.0 ± 16.6	0.271
Area of full thickness burn (%TBSA)	17.5 ± 28.4	19.9 ± 23.7	0.799
Crystalloids 1–24 hrs (L)	2.7 ± 0.7	2.7 ± 1.5	0.949
Colloids 1–24 hrs (L)	1.0 ± 0.6	0.7 ± 0.3	0.075
Total fluids 1–24 hrs (L)	5.5 ± 1.7	4.9 ± 2.1	0.329
Fluid rate 1–24 hrs (mL/hr)	307.9 ± 65.4	296.6 ± 132.0	0.731
Total urine 1–24 hrs (L)	1.2 ± 0.5	1.3 ± 0.6	0.682
Urine rate 1–24 hrs (mL/hr)	65.8 ± 29.9	75.3 ± 29.1	0.360
Crystalloids 25–48 hrs (L)	2.4 ± 0.8	2.1 ± 1.2	0.383
Colloids 25–48 hrs (L)	0.9 ± 0.5	0.8 ± 0.4	0.377
Total fluids 25–48 hrs (L)	5.5 ± 1.3	5.2 ± 1.8	0.320
Fluid rate 25–48 hrs (mL/hr)	239.2 ± 227.7	205.7 ± 80.5	0.320
Total urine 25–48 hrs (L)	1.9 ± 0.6	2.3 ± 0.8	0.088
Urine rate 25–48 hrs (mL/hr)	78.2 ± 24.1	95.4 ± 34.9	0.088
